# Supplementary material for: The Hamiltonian Brain: Efficient Probabilistic Inference with Excitatory-Inhibitory Neural Circuit Dynamics
Source: PLoS Comput Biol. 2016 Dec 27;12(12):e1005186. doi: 10.1371/journal.pcbi.1005186 (PMC5189947; doi:10.1371/journal.pcbi.1005186)
Supplement: S1 Fig — (PDF) [file pcbi.1005186.s001.pdf]

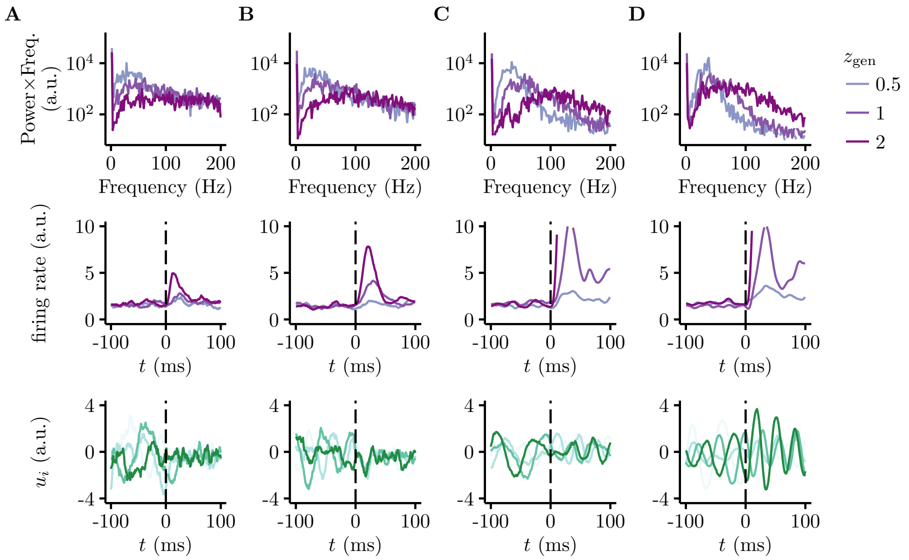

Figure S1. Our main results are robust to a range of ρ or equivalently τ_L. The top row is a power spectrum, the middle row displays the firing rate transient at stimulus onset, and the bottom row displays the membrane potential at stimulus onset for multiple trials and one neuron. The different lines in the first two rows correspond to different values of z_{gen}. In the bottom row, different lines correspond to different trials. **A.** For $\tau_L = 30$ ms, transients are small or non-existent, and no clear trends are present in the peak frequency. **B-C.** For $\tau_L = 60$ ms (**B**), and $\tau_L = 400$ ms (**C**) the results are similar to those in the main text. **D.** For $\tau_L = 1000$ ms, the results are quite different to those in the main text. In particular, the transient at stimulus onset lasts a long time, certainly longer than the observed value of around 50 ms.
